# The Association Between Kidney Disease and Mortality Among Adults With Cerebral Palsy—A Cohort Study: It Is Time to Start Talking About Kidney Health

**DOI:** 10.3389/fneur.2021.732329

**Published:** 2021-09-10

**Authors:** Daniel G. Whitney, Andrea L. Oliverio

**Affiliations:** ^1^Department of Physical Medicine and Rehabilitation, University of Michigan, Ann Arbor, MI, United States; ^2^Institute for Healthcare Policy and Innovation, University of Michigan, Ann Arbor, MI, United States; ^3^Division of Nephrology, Department of Internal Medicine, University of Michigan, Ann Arbor, MI, United States

**Keywords:** cerebral palsy, kidney disease, mortality, chronic kidney disease, clinical epidemiology

## Abstract

**Objective:** Recent evidence shows that adults with cerebral palsy (CP) have an increased risk for kidney disease, but nothing is known about how kidney disease integrates with their overall health. To begin understanding the importance of kidney health, the objective was to determine if kidney disease is associated with mortality among adults with CP after accounting for comorbidities common to CP and kidney disease.

**Methods:** Data from 2016 to 2018 from adults ≥18 years with CP were used from a random 20% sample fee-for-service Medicare database. Kidney disease in 2016 was ascertained as chronic kidney disease (CKD) stages 1–4, end stage kidney disease (ESKD), nephritic and nephrotic syndrome, and renal osteodystrophy. A modified version of the Whitney Comorbidity Index (modWCI) was used, which includes 24 comorbidities relevant to CP and kidney disease. Mortality rate ratio (MRR) through the year 2018 was estimated for each kidney disease and Cox regression estimated the hazard ratio (HR) of mortality after adjusting for demographics, co-occurring neurological conditions, and the modWCI.

**Results:** Prevalence of kidney disease was 7.3% among 16,728 adults with CP. MRR was elevated for any kidney disease (MRR = 3.14; 95%CI = 2.76–3.58) and most subtypes (MRR = 2.21–3.56; all *p* < 0.05). The adjusted HR of mortality remained elevated for any kidney disease (HR = 1.25; 95%CI = 1.09–1.45) and ESKD (HR = 1.38; 95%CI = 1.10–1.74).

**Discussion:** Kidney disease, especially ESKD, is associated with mortality among adults with CP independent of comorbidities that are relevant to CP and kidney disease. Findings suggest that nephrology care should be considered as part of routine clinical care for this population.

## Introduction

Cerebral palsy (CP) and kidney disease can have shared etiology, and the CP-related sequela can lead to or exacerbate kidney dysfunction. Pre-mature birth (1), stunted body and organ growth (2, 3), low activity and fitness levels (3, 4), altered body composition (5), and poor hydration status (6) are common among children with CP and are known risk factors for kidney disease. Furthermore, adults with CP have an increased risk for pre-mature development of hypertension, cardiometabolic diseases, and polypharmacy (7–11), which are well-established risk factors for chronic kidney disease (CKD) in the general population (12) and adults with CP (13, 14). Despite the shared etiologic and pathophysiologic pathways, little is actually known about kidney health in CP, especially as children with CP age into and throughout their adult years.

The burden of CKD is immense. Much of CKD is progressive, comes with a high degree of morbidity and mortality, and can lead to end stage kidney disease (ESKD). ESKD treatment and management is incredibly costly and time consuming, and can significantly hinder quality of life (15). Hemodialysis, the most common treatment for ESKD in the US, typically requires ~4 h per treatment, 3 days per week. Recent epidemiologic studies have begun to document just how common CKD and ESKD are for adults with CP. CKD stages 1–4 and ESKD were found to be 2.4-fold more prevalent for adults with vs. without CP (16). The 4-year incidence rate of advanced CKD (stages 4+) was 83% higher for adults with vs. without CP, which was not explained by their higher prevalence of cardiometabolic diseases (17). While these and other recent studies shed new light on the elevated risk of kidney disease and its risk factors unique to CP (13, 14, 16–18), nothing is known about how kidney disease integrates with overall health or influences unhealthful aging for adults with CP.

A grand challenge in developing clinical care pathways for adults with CP is understanding the appropriate timing for treatment and prioritization, due to the complexity of sorting through the array of diseases that pre-maturely afflict adults with CP (19). It is therefore unknown if treating and managing kidney disease should be prioritized over other health problems, which is in part complicated by clinical assessment of kidney function. For example, cardiovascular disease is 2.5-fold more prevalent for young adults with vs. without CP (20), and cardiovascular-related mortality is 3.2-fold higher for adults with vs. without CP (21). Kidney disease not only gives rise to or exacerbates cardiovascular disease, but many patients with CKD and ESKD will die from cardiovascular disease (22, 23).

However, as kidney disease is often clinically assessed by creatinine-based methods, the low muscle mass in CP leads to the erroneous interpretation that their kidney function is better than it is (18). This false negative can create a masked pathway of unhealthful aging, where the undetected kidney dysfunction over time may be driving, in part, the elevated cardiovascular-related morbidity and mortality burden in CP. Understanding the etiology of multimorbidity will help to develop more targeted prevention and treatment strategies, as well as clinical care guidelines for aging persons with CP.

A recent report found that kidney disease was associated with an increased risk of 2-year mortality among adults with CP (24). However, the association between kidney disease with mortality could not be examined independent of other comorbidities. This calls into question if kidney disease is independently associated with mortality among adults with CP beyond the influence of other comorbidities, or if kidney disease is a “downstream” consequence of aging with CP unrelated to mortality and benign to exacerbating multimorbidity profiles. Addressing this knowledge gap could assist in prioritizing clinical care for adults with CP. Therefore, the objective of this study was to determine if kidney disease is associated with mortality among adults with CP after accounting for comorbidities common to CP and kidney disease.

## Method

### Data Source

Data came from a 20% random sample from the Medicare fee-for-service administrative claims data source including the calendar years 2016–2018. Medicare is a U.S. federal program providing health insurance to adults ≥65 years of age or individuals of any age with ≥1 chronic disability, including CP, or ESKD. Administrative claims data are used for billing reimbursement from healthcare-related visits. Medical conditions can be identified by specific International Classification of Diseases, Tenth Revision, Clinical Modification (ICD-10-CM) codes that are attached to claims. A description of data ascertainment of this cohort, variables, and the ICD-10-CM codes used to identify each medical condition, as well as a flow chart of inclusion/exclusion criteria for this cohort have been reported previously (19, 24, 25). Data are de-identified and the University Institutional Review Board approved this study as non-regulated.

### Timeline and Sample Selection

The full calendar year 2016 was used to identify adults with CP ≥18 years of age (≥1 inpatient claim or ≥2 outpatient claims in 2016 for CP) that had continuous health plan enrollment in Part A and B from January 1, 2016 to January 30, 2017 to obtain a full 1-year baseline period and at least 30 days of follow-up for mortality, and without missing data for demographics. Data for all medical conditions and demographics were collected from the year 2016, and mortality data were collected from January 31, 2017 to December 31, 2018. The start date of follow-up for all participants was January 1, 2017. This allows for a 1-year baseline period and 2 years of follow-up for mortality.

### Mortality

All-cause mortality was ascertained as the number of days from January 31, 2017 to the date of death. Medicare has validated >99% of deaths (26). Participants that did not die during the follow-up were right-censored as the date of drop in health plan enrollment or the end of the study period (December 31, 2018)—whichever came first.

### Kidney Disease

Kidney disease was identified by CKD stages 1–4 (ICD-10-CM codes: I12.9, I13.0, I13.10, N18.1-N18.4); ESKD (including CKD stage 5), dialysis, or kidney transplant, hereafter referred to as “ESKD” (ICD-10-CM codes: I12.0, I13.11, I13.2, N18.5, N18.6, N19.x, Z49.x, Z94.0, Z99.2); CKD stage unspecified (ICD-10-CM code: N18.9); nephritic syndrome (ICD-10-CM codes: N03.x, N05.x); nephrotic syndrome (ICD-10-CM codes: N04.x); and renal osteodystrophy (ICD-10-CM code: N25.0). Nephritic and nephrotic syndrome and renal osteodystrophy are not mutually exclusive from one another and CKD or ESKD, but are examined separately in this study. Furthermore, renal osteodystrophy is a complication of CKD, but was included in this early phase of research to understand the association between kidney diseases and CP given the pervasive and profound bone fragility in this population (27, 28).

### Comorbidities

A modified version of the Whitney Comorbidity Index (WCI) was used to account for comorbidities relevant to CP and kidney disease. The WCI was recently developed in a privately insured cohort of adults with CP (24) and validated in this Medicare cohort with CP (25) and captures the unique morbidity profiles for adults with CP better than other commonly used comorbidity indices. The WCI is a single variable with a score ranging from 0 to 27 based on the number of WCI comorbidities present. However, the original WCI includes kidney disease, which was removed in this study as kidney disease is the exposure variable. Epilepsy and intellectual disabilities often co-occur with CP and may complicate the association between kidney disease and mortality. The WCI includes epilepsy and intellectual disabilities. However, these were removed from the modified version of the WCI and a new variable was constructed to isolate the effect of these co-occurring neurological conditions on the associations examined in this study. Specifically, mutually exclusive subgroups were assessed by stratifying the cohort as CP only, CP with co-occurring epilepsy but without intellectual disabilities (CP + EP), CP with co-occurring intellectual disabilities but without epilepsy (CP + ID), and CP + EP + ID. Therefore, the modified WCI in this study ranged from 0 to 24.

### Statistical Analysis

Baseline descriptive characteristics were summarized for the whole cohort with CP and for subgroups with or without kidney disease. Group differences between those with vs. without kidney disease for descriptive characteristics were tested using the Chi-square test for categorical variables and the independent *t*-test for continuous variables.

Crude mortality rate (MR) with 95% confidence intervals (CI) was estimated for any kidney disease and the specific conditions (e.g., CKD stages 1–4, renal osteodystrophy) separately as the number of deaths divided by the number of person-years, and expressed as per 100 person years. Crude MR ratio (MRR with 95% CI) was estimated for any kidney disease and the specific conditions separately using the group without that kidney disease as the reference. Cox proportional hazards regression models were developed to estimate the hazard ratio (HR with 95% CI) of mortality for any kidney disease and the specific conditions after adjusting for two groups of covariates: model 1—age (as continuous), sex, race, U.S. region of residence, and co-occurring epilepsy and/or intellectual disabilities; model 2—model 1 covariates plus the modified WCI. Clinically relevant interactions were examined for each exposure variable with age, sex, race, and co-occurring epilepsy and/or intellectual disabilities by separate analyses for the main effect and including the product term in the Cox model.

Analyses were performed using SAS version 9.4 (SAS Institute, Cary, NC, USA) and *p* ≤ 0.05 (two-tailed) was used to determine statistical significance.

## Results

Of the 16,728 adults with CP, 7.3% (*n* = 1,215) had kidney disease, with the majority having any stage of CKD (7.2%) and 2.0% having ESKD. To put that into perspective, the prevalence in 2015 of ESKD for the general population ≥45 years of age was ~0.5% (29).

Baseline descriptive characteristics for the whole cohort with CP (*n* = 16,728) and for the subgroups without (*n* = 15,513) and with (*n* = 1,215) kidney disease are presented in Table 1. Adults with CP with kidney disease were on average 10.1 years older, had a slightly higher proportion of Black adults and CP only, and a higher modified WCI score that was more than double on average (7.2 vs. 3.5) compared to adults with CP without kidney disease (all *p* < 0.01). Adults with CP with vs. without kidney disease had a higher prevalence of all comorbidities in the modified WCI (*p* < 0.001), except for the lower prevalence of epilepsy and intellectual disabilities (*p* < 0.05; Supplementary Table 1).

**Table 1:** Baseline descriptive characteristics and prevalence of kidney disease for adults with cerebral palsy (CP) with or without kidney disease.

	**Entire group**	**CP without kidney disease**	**CP with kidney disease**
	**(*n* = 16,728)**	**(*n* = 15,513)**	**(*n* = 1,215)**
**Descriptive characteristics**			
Age, mean (SD)	51.0 (15.3)	50.3 (15.1)	60.4 (14.9)^*^
18–40 years, % (*n*)	27.7 (4,633)	29.0 (4,491)	11.7 (142)
41–64 years, % (*n*)	51.6 (8,631)	52.0 (8,067)	46.4 (564)
≥65 years, % (*n*)	20.7 (3,464)	19.0 (2,955)	41.9 (509)
**Sex, % (** ***n*** **)**			
Male	51.8 (8,662)	51.6 (8,001)	54.4 (661)
Female	48.2 (8,066)	48.4 (7,512)	45.6 (554)
Race, % (*n*)			^*^
White	80.5 (13,471)	80.5 (12,492)	80.6 (979)
Black	13.0 (2,180)	12.9 (1,993)	15.4 (187)
Hispanic	3.4 (560)	3.5 (535)	2.1 (25)
Asian	1.0 (167)	1.0 (162)	0.4 (5)
North American Native	0.9 (142)	0.9 (137)	0.4 (5)
Other	1.2 (208)	1.3 (194)	1.2 (14)
**U.S. region of residence, % (** ***n*** **)**			
Midwest	27.7 (4,639)	27.7 (4,292)	28.6 (347)
Northeast	21.7 (3,631)	21.9 (3,399)	19.1 (232)
South	34.4 (5,756)	34.3 (5,313)	36.5 (443)
West	16.2 (2,702)	16.2 (2,509)	15.9 (193)
**Co-occurring neurological conditions**			^*^
CP only	45.1 (7,542)	44.7 (6,927)	50.6 (615)
CP + EP	15.6 (2,607)	15.7 (2,433)	14.3 (174)
CP + ID	16.6 (2,781)	16.8 (2,610)	14.1 (171)
CP + EP + ID	22.7 (3,798)	22.8 (3,543)	21.0 (255)
Modified Whitney comorbidity index			^*^
Mean (SD)	4.5 (3.2)	3.5 (2.8)	7.2 (3.6)
Median (IQR)	4.0 (2–6)	3 (1–5)	7 (4–10)
**Kidney disease, % (** ***n*** **)**			
Any kidney disease	7.3 (1,215)		
Chronic kidney disease (CKD)	7.2 (1,202)		
CKD stages 1–4	4.3 (712)		
End stage kidney disease (including CKD stage 5)	2.0 (333)		
CKD stage unspecified	0.9 (157)		
Nephritic syndrome	0.2 (25)		
Nephrotic syndrome	0.1 (10)		
Renal osteodystrophy	0.2 (25)		

*SD, standard deviation; IQR, interquartile range*.

* *p < 0.01 compared to adults with CP without kidney disease*.

During the 2-year follow-up for a mean (SD) of 696 (126) days and a median [interquartile range (IQR)] of 730 (730–730) days, a total of 1,486 (8.9%) died with a mean (SD) age at death of 61.9 (15.0) years, while 27 (0.1%) were right-censored owing to a drop in health plan enrollment and 15,215 (91.0%) were right-censored owing to being alive by the end of the study period. The follow-up time was similar for the subgroups: without kidney disease, 7.8% (1,213) died with a mean (SD) age at death of 61.0 (15.1) years; with kidney disease, 273 (22.5%) died with a mean (SD) age at death of 65.5 (13.9) years.

### Association Between Kidney Disease and Mortality

**Figure 1:**
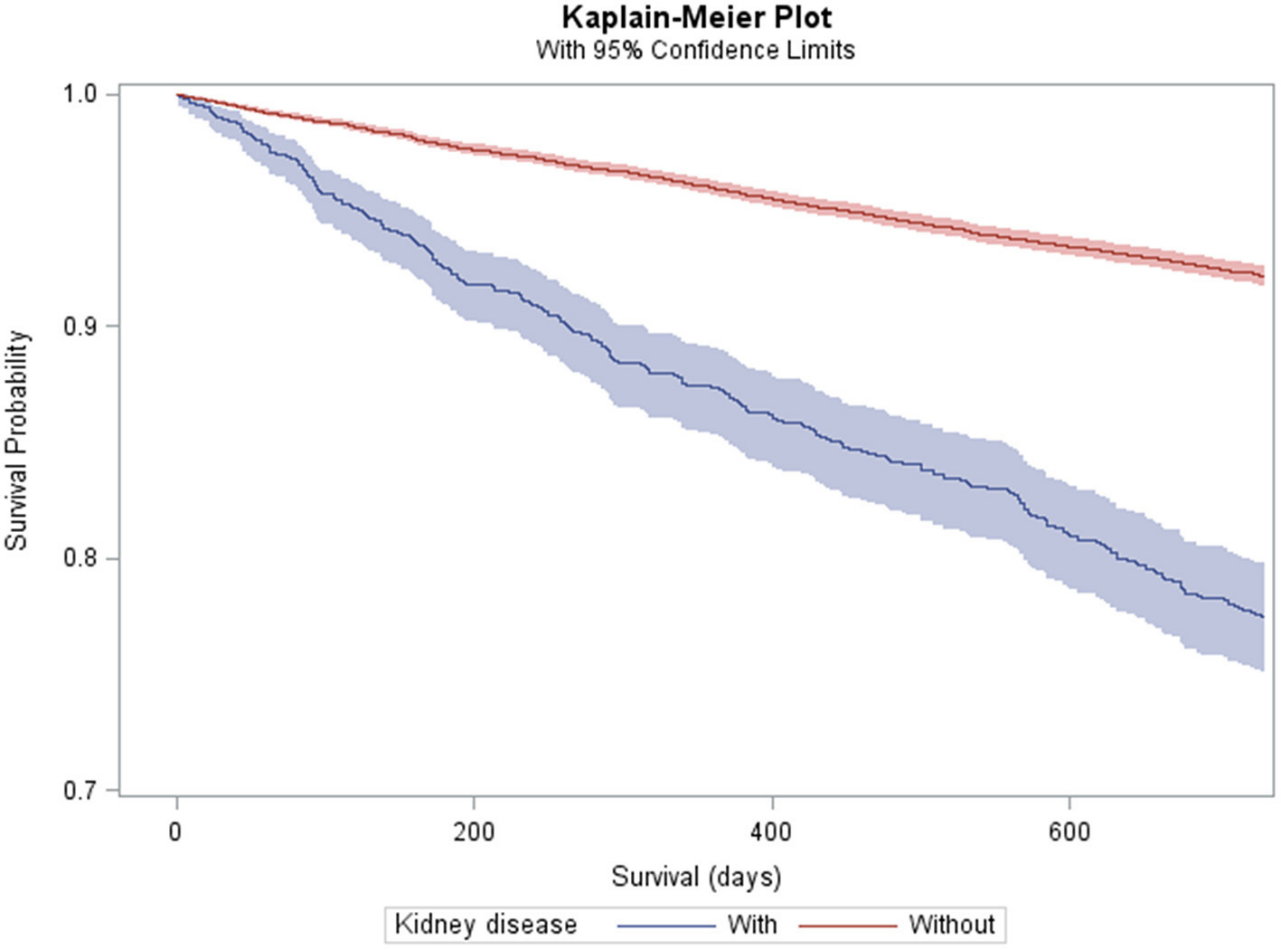
Kaplan–Meier plot and 95% confidence limits (shaded area) of mortality among adults with cerebral palsy with (*n* = 1,215) and without (*n* = 15,513) kidney disease.

Any kidney disease (Figure 1) and each subtype was associated with an elevated crude MR and MRR (Table 2). After adjusting for all covariates, any kidney disease was associated with an elevated HR of mortality (HR = 1.25; 95% CI = 1.09–1.45) (Table 2). Among the specific conditions, ESKD was associated with an elevated HR of mortality (HR = 1.38; 95% CI = 1.10–1.74), as was CKD stages 1–4, but this was not statistically significant (HR = 1.16; 95% CI = 0.98–1.38; *p* = 0.092). The other specific conditions had too few outcome cases to perform adjusted analyses.

**Table 2:** Mortality rate (MR), MR ratio (MRR)^*^, and adjusted hazard ratio (HR)^*^ of mortality for each kidney disease among adults with cerebral palsy (*n* = 16,728).

	**Mortality cases (*n*)**	**MR per 100 person years (95% CI)**	**MRR (95% CI)**	**Model 1 HR (95% CI)**	**Model 2 HR (95% CI)**
Any kidney disease	273	12.8 (11.3, 14.3)	3.14 (2.76, 3.58)	2.04 (1.78, 2.34)	1.25 (1.09, 1.45)
CKD 1–4	156	12.4 (10.5, 14.4)	2.87 (2.43, 3.38)	1.72 (1.45, 2.04)	1.16 (0.98, 1.38)
ESKD	86	15.1 (11.9, 18.3)	3.38 (2.72, 4.20)	2.54 (2.04, 3.16)	1.38 (1.10, 1.74)
CKD stage unspecified	29	10.2 (6.5, 13.9)	2.21 (1.53, 3.20)	1.54 (1.06, 2.22)	^**^
Nephritic syndrome	7	16.6 (4.3, 28.8)	3.56 (1.70, 7.49)	^**^	^**^
Nephrotic syndrome	2	^**^	^**^	^**^	^**^
Renal osteodystrophy	6	13.9 (2.8, 25.0)	2.99 (1.34, 6.68)	^**^	^**^

*CI, confidence interval; CKD, chronic kidney disease; ESKD, end stage kidney disease*.

* *Reference is the group without that disease; e.g., reference for any kidney disease includes those without any kidney disease*.

** *Too few mortality cases for analysis*.

*Model 1 adjusted for: age (continuous), sex, race, U.S. region of residence, and co-occurring epilepsy and intellectual disabilities. Model 2 adjusted for: model 1 covariates and the modified Whitney Comorbidity Index*.

### Interactions

While there was an interaction between ESKD and race (*p* for interaction, <0.001) and between ESKD and age (*p* for interaction, 0.001), the crude MR was similar between White and Black adults with CP with ESKD (interaction due to racial difference for adults with CP without ESKD) (Table 3). There were too few mortality cases to perform analyses for adults with ESKD that were Hispanic (*n* = 2), Asian (*n* = 2), North American Native (*n* = 0), and other race (*n* = 2). While the crude MR was higher for older age groups, the relative mortality risk (i.e., MRR) was higher for younger age groups when age was stratified as young, middle-aged, and elderly (Table 3).

**Table 3:** Mortality rate (MR), MR ratio (MRR), and adjusted hazard ratio (HR)^*^ of mortality for those with and without end stage kidney disease (ESKD) by race and age group.

	**Mortality, % (*n*)**	**MR per 100 person years (95% CI)**	**MRR (95% CI)**	**HR (95% CI)**
**Race**				
**White (** ***n*** **=** **13,471)**				
Without ESKD	9.1 (1,196)	4.8 (4.5, 5.0)	Reference	Reference
With ESKD	25.3 (64)	14.8 (11.1, 18.4)	3.11 (2.42, 3.99)	1.29 (0.99, 1.68)
**Black (** ***n*** **=** **2,180)**				
Without ESKD	6.4 (136)	3.3 (2.8, 3.9)	Reference	Reference
With ESKD	25.0 (16)	14.5 (7.4, 21.6)	4.35 (2.59, 7.31)	^**^
**Age**				
**Young, 18–40 years (** ***n*** **=** **4,633)**				
Without ESKD	3.0 (135)	1.5 (1.3, 1.8)	Reference	Reference
With ESKD	13.8 (8)	7.4 (2.3, 12.6)	4.95 (2.42, 10.10)	^**^
**Middle-aged, 41–64 years (** ***n*** **=** **8,631)**				
Without ESKD	7.3 (614)	3.8 (3.5, 4.1)	Reference	Reference
With ESKD	26.5 (45)	15.5 (10.9, 20.0)	4.10 (3.03, 5.54)	1.83 (1.32, 2.54)
**Elderly**, **≥65 years (*****n*****=3,464)**				
Without ESKD	18.8 (651)	10.8 (10.0, 11.6)	Reference	Reference
With ESKD	31.4 (33)	19.4 (12.8, 26.0)	1.80 (1.27, 2.56)	0.92 (0.64, 1.33)

*CI, confidence interval*.

* *Model adjusted for age (continuous), sex, race (only for the age group analysis), U.S. region of residence, co-occurring epilepsy and intellectual disabilities, and the modified Whitney Comorbidity Index*.

** *Too few mortality cases for analysis*.

## Discussion

This study found that kidney disease among adults with CP, especially ESKD, was associated with mortality above the influence of other comorbidities relevant to CP and kidney disease. Furthermore, while the mortality rate was higher for older adults, the relative mortality risk was higher for younger adults with CP with vs. without ESKD. The latter finding may be due to the higher mortality rate observed among individuals with more vs. less severe forms of CP, who also have an earlier and elevated burden of disease (30).

Inconsistent with the general population (23), this study found that Black and White adults with CP and ESKD had a similar MR (14.5 and 14.8, respectively). A previous study found that race and sex were not associated with incidence of advanced stages of CKD among adults with CP (13), which is also inconsistent with the general population (12). The mechanisms for the lack of race and sex effects on CKD risk and the associated mortality is not clear. One possible explanation is that the biological, socioeconomic, and health disparity differences between race and sex strata may be less disparate in the context of CP, possibly due to the complex unhealthful aging process “over-riding” any biological and/or environmental or social differences that would arise from race and sex, although more research is needed.

The clinical challenge for the field is not so much wrestling with the notion that kidney disease can be deadly for adults with CP, which is well-known in the general population, but rather, a lack of awareness that kidney disease may be a pervasive problem carrying a high mortality risk for adults with CP, which may be masked by the high cardiovascular-related mortality burden (21). Primary care physicians, neurologists, and rehabilitation clinicians are typically the clinical disciplines that frequently interface with adults with CP for their healthcare management. Unfortunately, many clinicians are unaware of the medical complexities of aging with CP, or how to interpret clinical tests that may be altered in CP for appropriate treatment or medical referrals, leading to suboptimal care (31). For example, routine blood work estimates glomerular filtration rate (eGFR), a marker of kidney function, using serum creatinine and equations established from samples without CP. Due to the low muscle mass in CP resulting in low creatinine, the creatinine-based method overestimates eGFR, leading to the interpretation that kidney function is higher than it actually is (18). Clinicians that treat adults with CP are generally unaware of this issue. The clinical consequence is a missed opportunity for a nephrology referral, or early recognition and intervention by primary care physicians to reduce the risk of CKD progression (e.g., controlling hypertension, limiting nephrotoxic drugs). This calls into question just how common and deadly CKD actually is for adults with CP, which may be underestimated in previous studies (13, 14, 17) and this study.

Cystatin c is another biomarker that can be used for eGFR (32), and is less impacted by age, sex, diet, and muscle mass (33). In a non-CP cohort with significant muscle deficits, cystatin c-based eGFR was a better predictor of kidney function than creatinine-based eGFR (34). Although since cystatin c is expressed by adipose tissue (35), more work is needed to identify whether cystatin c or creatinine may be a better biomarker to estimate kidney function in populations with low muscle and high body and regional fat stores, such as CP (3, 5, 36, 37).

Nephrologists are aware of the issues in interpreting eGFR and assessing kidney function in the context of low muscle mass conditions, such as CP. However, nephrology is currently not a routine part of clinical care for individuals with CP at any age. Importantly, advanced stages of CKD are irreversible, costly, and fatal (15), so delaying the onset of CKD and mitigating the progression to advanced stages is essential. Therefore, while more work is needed to deliver targeted clinical recommendations, we urge the clinical community to consider nephrology referrals whenever feasible, which may serve at least as preventive care, so that adults with CP can get the provision of services they may need. We also recommend utilizing albuminuria values in addition to eGFR for CKD prognosis when determining a nephrology referral, as outlined by the KDIGO guidelines (Kidney Disease: Improving Global Outcomes) (38).

It is important to note that CKD and ESKD appeared in this study as the more pervasive kidney issues. While nephritic and nephrotic syndrome and renal osteodystrophy appeared to be rare in this cohort, we felt it was necessary to initially examine these non-CKD kidney conditions, to determine future directions; i.e., whether to focus future research efforts into CKD and/or other kidney conditions, although some of the non-CKD kidney conditions examined in this study can be acute and comorbid with CKD.

This study has identified for the first time an independent association between kidney disease and mortality in adults with CP; yet, there remains a number of questions in regard to how integrative and deleterious kidney disease is for aging and quality of life for this population. For example, does kidney disease lead to or exacerbate cardiovascular-related morbidity and mortality among adults with CP? Are there distinct etiologies of kidney disease for different segments of the population with CP, such as those born pre-mature, or those with normal birth but develop cardiometabolic disease early in life? If there are different etiologies of kidney disease in CP, does clinical care differ in its approach to prevention and treatment? To address these and other questions, studies are first needed to identify new methods or refinements to existing methods that more accurately capture kidney function for adults with CP (18), which will need to account for the severity of CP.

The limitations of this study must be discussed. First, claims data do not contain information on how diagnoses were made or by whom (e.g., nephrologist). It is possible that many adults with CP that have kidney disease are not being diagnosed, many of which may have died in the current study, leading to conservative estimates of mortality risk associated with kidney disease. Second, each kidney disease was examined separately for those with vs. without that condition, which may have led to more conservative estimates. For example, those with ESKD were compared to those without ESKD, but the non-ESKD group included those with CKD stages 1–4 and the other specific kidney conditions. Third, this study examined prevalent rather than incident kidney disease, which does not provide a sense of the time-related factor of having kidney disease and its impact on organ-specific or overall pathophysiology. It is difficult to make an assumption of how this impacts the mortality risk estimates in this study in conjunction with the first two stated limitations. Fourth, claims data do not contain information on the cause of death. Fifth, severity of CP cannot be reliably ascertained in claims data. Therefore, study findings can be viewed as empirical evidence to contribute to a more general discussion on the need for more nephrology services and research for adults with CP as a whole. However, the WCI estimates the overall disease status, and can be a proxy of severity of CP, which was adjusted for in the analyses. This provides some indication that findings may be present across the CP severity spectrum, but still likely differs in the magnitude and timing by severity of CP. Lastly, as data come from a single, nationwide insurance database, although large, findings may not be generalizable to all adults with CP.

In conclusion, kidney disease is associated with an increased risk of mortality for adults with CP, independent of other comorbidities relevant to CP and kidney disease. Findings provide preliminary evidence for the need to consider incorporating nephrology care as part of routine clinical care for adults with CP. Future research is needed to determine if adopting nephrology care as part of routine clinical care for adults with CP improves detection, increases preventive strategies, and improves kidney and overall health for this underserved population.

## Data Availability Statement

The data analyzed in this study was obtained from the Centers for Medicare and Medicaid Services, the following licenses/restrictions apply: The dataset analyzed in this study may be accessed through a contractual agreement with the Centers for Medicare and Medicaid Services following the payment of an administrative fee. Information about these datasets and requests for dataset access can be found at: https://www.cms.gov/Research-Statistics-Data-and-Systems/Research-Statistics-Data-and-Systems.

## Ethics Statement

The studies involving human participants were reviewed and approved by University of Michigan Institutional Review Board. Written informed consent for participation was not required for this study in accordance with the national legislation and the institutional requirements.

## Author Contributions

DW analyzed the data and wrote the first draft of the manuscript. DW and AO conceptualized, designed the study, approve the final version of this manuscript, and agree to be accountable for the content of the work. AO edited the manuscript. All authors contributed to the article and approved the submitted version.

## Funding

This work was supported by the University of Michigan Office of Health Equity and Inclusion Diversity Fund (DW). The funding source had no role in the design and conduct of the study; collection, management, analysis, and interpretation of the data; preparation, review, or approval of the manuscript; and decision to submit the manuscript for publication.

## Conflict of Interest

The authors declare that the research was conducted in the absence of any commercial or financial relationships that could be construed as a potential conflict of interest.

## Publisher's Note

All claims expressed in this article are solely those of the authors and do not necessarily represent those of their affiliated organizations, or those of the publisher, the editors and the reviewers. Any product that may be evaluated in this article, or claim that may be made by its manufacturer, is not guaranteed or endorsed by the publisher.
