# Supplementary material for: The Association Between Kidney Disease and Mortality Among Adults With Cerebral Palsy—A Cohort Study: It Is Time to Start Talking About Kidney Health
Source: Front Neurol. 2021 Sep 10;12:732329. doi: 10.3389/fneur.2021.732329 (PMC8460853; doi:10.3389/fneur.2021.732329)
Supplement: Supplementary file 1 [file Table_1.DOCX]

**Supplementary Table 1.** Prevalence of individual comorbidities from the modified Whitney Comorbidity Index for adults with cerebral palsy (CP) with or without kidney disease.

|  | CP without kidney disease  (n=15,513) | CP with kidney disease  (n=1,215) |
| --- | --- | --- |
|  | % (n) | % (n) |
| Hypertension (Un)complicated | 41.8 (6,488) | 85.8 (1,042) *** |
| Intellectual disabilities | 39.7 (6,153) | 35.1 (426) ** |
| Epilepsy | 38.5 (5,976) | 35.3 (429) * |
| Other neurological disorders | 35.0 (5,426) | 46.1 (560) *** |
| Depression | 27.3 (4,229) | 40.6 (493) *** |
| Fluid and electrolyte disorders | 22.8 (3,540) | 59.2 (719) *** |
| Gastrointestinal issues | 23.6 (3,653) | 27.4 (333) ** |
| Dysphagia | 20.0 (3,098) | 32.2 (391) *** |
| Hypothyroidism | 19.7 (3,060) | 33.3 (405) *** |
| Bone fragility | 20.3 (3,154) | 25.9 (315) *** |
| Osteoarthritis and allied disorders | 18.5 (2,862) | 34.3 (417) *** |
| Cardiac arrhythmias | 16.7 (2,587) | 42.0 (510) *** |
| Diabetes without chronic complication | 14.0 (2,172) | 40.7 (495) *** |
| Pneumonia | 13.3 (2,064) | 32.5 (395) *** |
| Chronic pulmonary disease | 12.6 (1,961) | 30.6 (372) *** |
| Blood loss and deficiency anemias | 11.1 (1,721) | 32.8 (399) *** |
| Cerebrovascular disease | 8.7 (1,349) | 23.4 (284) *** |
| Diabetes with chronic complication | 7.3 (1,131) | 32.5 (395) *** |
| Congestive heart failure | 6.5 (1,009) | 31.7 (385) *** |
| Neurogenic bowel or bladder | 6.9 (1,076) | 15.0 (182) *** |
| Dementia | 5.6 (874) | 16.4 (199) *** |
| Any malignancy, including lymphoma and leukemia, except malignant neoplasm of skin | 5.5 (853) | 10.9 (132) *** |
| Mild to severe liver disease | 4.5 (693) | 8.7 (106) *** |
| Myocardial infarction | 1.7 (269) | 10.0 (122) *** |
| Rheumatoid arthritis and other inflammatory polyarthropathies | 1.5 (226) | 3.7 (45) *** |
| Metastatic cancer | 0.6 (98) | 2.1 (26) *** |

**P*<0.05, ** *P*<0.01, *** *P*<0.001 compared to adults with CP without kidney disease.
